# Living with pathological narcissism: a qualitative study

**DOI:** 10.1186/s40479-020-00132-8

**Published:** 2020-08-14

**Authors:** Nicholas J. S. Day, Michelle L. Townsend, Brin F. S. Grenyer

**Affiliations:** Illawarra Health and Medical Research Institute and School of Psychology, University of Wollongong Australia, Wollongong, NSW Australia

**Keywords:** Narcissistic personality disorder, Pathological narcissism, Personality disorder, Grandiosity, Vulnerability, Interpersonal functioning, Qualitative research

## Abstract

**Background:**

Research into the personality trait of narcissism have advanced further understanding of the pathological concomitants of grandiosity, vulnerability and interpersonal antagonism. Recent research has established some of the interpersonal impacts on others from being in a close relationship with someone having such traits of pathological narcissism, but no qualitative studies exist. Individuals with pathological narcissism express many of their difficulties of identity and emotion regulation within the context of significant interpersonal relationships thus studying these impacts on others is warranted.

**Method:**

We asked the relatives of people high in narcissistic traits (indexed by scoring above a cut-off on a narcissism screening measure) to describe their relationships (*N* = 436; current romantic partners [56.2%]; former romantic partners [19.7%]; family members [21.3%]). Participants were asked to describe their relative and their interactions with them. Verbatim responses were thematically analysed.

**Results:**

Participants described ‘grandiosity’ in their relative: requiring admiration, showing arrogance, entitlement, envy, exploitativeness, grandiose fantasy, lack empathy, self-importance and interpersonal charm. Participants also described ‘vulnerability’ of the relative: contingent self-esteem, hypersensitivity and insecurity, affective instability, emptiness, rage, devaluation, hiding the self and victimhood. These grandiose and vulnerable characteristics were commonly reported together (69% of respondents). Participants also described perfectionistic (anankastic), vengeful (antisocial) and suspicious (paranoid) features. Instances of relatives childhood trauma, excessive religiosity and substance abuse were also described.

**Conclusions:**

These findings lend support to the importance of assessing the whole dimension of the narcissistic personality, as well as associated personality patterns. On the findings reported here, the vulnerable aspect of pathological narcissism impacts others in an insidious way given the core deficits of feelings of emptiness and affective instability. These findings have clinical implications for diagnosis and treatment in that the initial spectrum of complaints may be misdiagnosed unless the complete picture is understood. Living with a person with pathological narcissism can be marked by experiencing a person who shows large fluctuations in affect, oscillating attitudes and contradictory needs.

## Introduction

The current diagnostic description of narcissistic personality disorder (NPD) as it appears in the *Diagnostic and Statistical Manual of Mental Disorders* (DSM-5, 5th edition, [1]) includes a lot of information about how the person affects others, such as requiring excessive admiration, having a sense of entitlement, interpersonal exploitativeness, showing both a lack of empathy for others and feeling others are envious of their perceived special powers or personality features. Despite these features being important aspects of narcissism that have been validated through empirical research [2, 3], they have been criticised for their emphasis on grandiosity and the exclusion of vulnerability in narcissism [4, 5], a trend that is mirrored in the field more generally and runs counter to over 35 years of clinical theory [3]. The more encompassing term ‘pathological narcissism’ has been used to better reflect personality dysfunction that is fundamentally narcissistic but allows for both grandiose and vulnerable aspects in its presentation [6].

Recognising the vulnerable dimension of narcissism has significant implications for treatment [7], including providing an accurate diagnosis and implementing appropriate technical interventions within treatment settings. Vulnerable narcissism, in marked contrast to the overt grandiose features listed in DSM-5 criteria, includes instances of depressed mood, insecurity, hypersensitivity, shame and identification with victimhood [8–12]. Pincus, Ansell [13] developed the Pathological Narcissism Inventory (PNI) to capture this narcissistic vulnerability in three factors. The factor ‘contingent self-esteem’ (item example: ‘It’s hard for me to feel good about myself unless I know other people like me’) reflects a need to use others in order to maintain self-esteem. The factor ‘devaluing’ includes both devaluation of others who do not provide admiration needs (‘sometimes I avoid people because I’m concerned that they’ll disappoint me’) and of the self, due to feelings of shameful dependency on others (‘when others disappoint me, I often get angry at myself’). The factor ‘hiding the self’ (‘when others get a glimpse of my needs, I feel anxious and ashamed’) reflects an unwillingness to show personal faults and needs. This factor may involve a literal physical withdrawal and isolation [14] but may also include a subtler emotional or psychic withdrawal due to feelings of inadequacy and shame which may result in the development of an imposter or inauthentic ‘false self’ [11, 15], and which may also include a disavowal of emotions, becoming emotionally ‘empty’ or ‘cold’ [14]. Another aspect described in the literature are instances of ‘narcissistic rage’ [16] marked by hatred and envy in response to a narcissistic threat (i.e. threats to grandiose self-concept). Although commonly reported in case studies and clinical reports, it is unclear if it is a feature of only grandiose presentations or if it may more frequently present in vulnerable presentations [17].

While the differences in presentation between grandiose and vulnerable narcissism appear manifest, it has been argued that they reflect both sides of a narcissistic ‘coin’ [9] that may be regularly oscillating, inter-related and state dependent [6, 18–22]. As such, it may not be as important to locate the specific presentation of an individual as to what ‘type’ they are (i.e. grandiose or vulnerable), as it is to recognise the presence of *both* of these aspects within the person [23]. The difficulty for these patients is the pain and distress that accompanies having such disparate ‘split off’ or unintegrated parts of the self, which result in the defensive use of maladaptive intra and interpersonal methods of maintaining a stable self-experience [24]. This defensive operation is somewhat successful, and may give the impression of a coherent and stable identity, however as noted by Caligor and Stern [25] “manifestly vulnerable narcissists retain a connection to their grandiosity … [and] even the most grandiose narcissist may have internal feelings of inadequacy or fraudulence” (p. 113).

The vulnerable dimension of narcissism, with its internal feelings of emptiness and emotion dysregulation, may reflect a more general personality pathology similar to that of borderline personality disorder (BPD) [26]. For instance, Euler, Stobi [27] found grandiose narcissism to be related to NPD, but vulnerable narcissism to be related to BPD. In a similar vein, Hörz-Sagstetter, Diamond [28] proposes grandiosity as a narcissistic ‘specific’ factor that distinguishes it from other disorders (e.g. BPD). This grandiosity, however, *“*predisposes [these individuals] to respond with antagonism/hostility and reduced reality testing when the grandiose self is threatened*”* (p.571). This antagonism, hostility and the resultant interpersonal dysfunction are well-documented aspects of pathological narcissism [29–32], that exacts a large toll on individuals in the relationship [33, 34]. As the specific features of the disorder are perhaps therefore best evidenced within the context of these relationships, gaining the perspective of the ‘other’ in the relationship would present a unique perspective that may not be observable in other contexts (e.g. clinical or self-report research). For example, a recent study by Green and Charles [35] provided such a perspective within the context of domestic violence. They found that those in a relationship with individuals with reportedly narcissistic features described overt (e.g. verbal and physical) and covert (e.g. passive-aggressive and manipulative) expressions of abuse and that these behaviours were in response to perceived challenges to authority and to counteract fears of abandonment. As such, informant ratings may be a novel and valid methodology to assess for personality pathology [36], as documented discrepancies between self-other ratings suggest that individuals with pathological narcissism may not provide accurate self-descriptions [37]. Further, Lukowitsky and Pincus [38] report high levels of convergence for informant ratings of narcissism, indicating that multiple peers are likely to score the same individual similarly and, notably, individuals with pathological narcissism agreed with observer ratings of interpersonal dysfunction, again highlighting this aspect as central to the disorder [6]. The aim of this study is to investigate the reported characteristics of individuals with pathologically narcissistic traits from the perspective of those in a significant personal relationship with these individuals. For this research, partners and family members will be referred to as ‘participants’. Individuals with pathological narcissism will be referred to as the ‘relative’.

## Method

### Recruitment

Participants were relatives of people reportedly high in narcissistic traits, and all provided written informed consent to allow their responses to be used in research, following institutional review board approval. The participants were recruited through invitations posted on various mental health websites that provide information and support that is narcissism specific (e.g. ‘Narcissistic Family Support Group’). Recruitment was advertised as being specifically in relation to a relative with narcissistic traits. A number of criteria were applied to ensure that included participants were appropriate to the research. First, participants had to identify as having a ‘significant personal relationship’ with their relative. Second, participants had to complete mandatory questions as part of the survey. Mandatory questions included basic demographic information (age, gender, relationship type) and answers to qualitative questions under investigation. Non-mandatory questions included questions such as certain demographic questions (e.g. occupation) and questions pertaining to their own support seeking. Third, the relative had to have a cumulative score of 36 (consistent with previous methodology, see [33]) or above on a narcissism screening measure (described in Measures section), as informed by participants.

### Participants

A total of 2219 participants consented to participate in the survey. A conservative data screening procedure was implemented to ensure that participants were appropriate to the research. First, participants were removed who indicated that they did not have a ‘significant’ (i.e. intimate) personal relationship with someone who was narcissistic (*n* = 129). Second, participants who clicked on the link to begin the survey but dropped out within the first 1–5 questions were deemed ‘non-serious’ and were removed (*n* = 1006). Third, participants whose text sample was too brief (i.e. less than 70 words) to analyse were excluded (*n* = 399) as specified by Gottschalk, Winget [39]. Finally, participants identified as rating relatives narcissism below cut off score of 36 on a narcissism screening measure were removed (*n* = 249). Inspection of pattern of responses indicated that none of the remaining participants had filled out the survey questions inconsistently or inappropriately (e.g. scoring the same for all questions). The remaining 436 participants formed the sample reported here. Table 1 outlines the demographic information of participants and the relative included in the study.

**Table 1:** Demographics for participants (partners and family) and their relatives (people high in pathological narcissism) (*N* = 436)

	Participants(*n* = 436)	Relative (*n* = 436)
Mean age in years (SD)	43.7 (10.1)	48.7 (12.3)
Gender		
Male	4.8% (*n* = 21)	75.7% (*n* = 330)
Female	79.6% (*n* = 347)	24.3% (*n* = 106)
Not Specified	15.6% (*n* = 68)	–
Employment		
Full time	42.7% (*n* = 186)	50.7% (*n* = 221)
Part time	14.9% (*n* = 65)	8.3% (*n* = 36)
Unemployed	9.9% (n = 43)	12.4% (*n* = 54)
Other	32.6% (*n* = 142)	28.7% (*n* = 125)
Disability Pension	3.2% (n = 14)	4.4% (*n* = 19)
Self-Employed	3.7% (*n* = 16)	9.9% (*n* = 43)
Retired	3.4% (n = 15)	8.9% (n = 39)
Student	2.1% (*n* = 9)	0.2% (n = 1)
Not stated	20.2% (*n* = 88)	5.3% (*n* = 23)
Relationship		
Spouse/partner	56.2%, (*n* = 245)	
Former spouse/partner	19.7%, (*n* = 86)	
Family (total)	21.3% (*n* = 93)	
Mother	10.6% (*n* = 46)	
Father	2.5% (*n* = 11)	
Child	1.4% (n = 6)	
Sibling	4.1% (n = 18)	
Other Family	2.8% (n = 12)	
Other	2.8% (n = 12)	

Participants were also asked to report on the diagnosis that their relative had received. These diagnoses were specified as being delivered by a mental health professional and not the participants own speculation. The majority of participants either stated that their relative has not received a formal diagnosis, or that they did not know (*n* = 284, 65%). A total of 152 (35%) participants stated that their relative had received an official diagnosis from a mental health professional (See Table 2).

**Table 2:** Relatives diagnoses as reported by participants (*n* = 152)

Personality disorder	43% (*n* = 65)
Narcissistic Personality Disorder	29% (*n* = 44)
Borderline Personality Disorder	5% (*n* = 9)
Other	7% (*n* = 11)
Not Specified	4% (*n* = 7)
Attention Deficit-Hyperactivity Disorder	12% (*n* = 18)
Anxiety Related Disorder	10% (*n* = 15)
Obsessive-Compulsive Related Disorder	7% (*n* = 10)
Substance Related and Addictive Disorders	5% (*n* = 8)
Bipolar and Related Disorders	20% (*n* = 31)
Depressive Disorders	30% (*n* = 46)
Autism Spectrum Disorders	1% (*n* = 2)
Trauma Related Disorders	9% (*n* = 14)
Psychotic Disorders	5% (*n* = 7)

*Note*. The percentages and numbers of diagnoses endorsed are greater than the total number of participants as many relatives had been diagnosed with ‘co-morbid’ disorders. ‘Other’ personality disorder group includes avoidant (*n* = 3), histrionic (*n* = 2), antisocial (*n* = 4), schizoid (*n* = 1) and paranoid (*n* = 1)

### Measures

#### Pathological narcissism inventory (Carer version) (SB-PNI-CV)

Schoenleber, Roche [40] developed a short version of the Pathological Narcissism Inventory (SB-PNI; ‘super brief’) as a 12 item measure consisting of the 12 best performing items for the Grandiosity and Vulnerability composites (6 of each) of the Pathological Narcissism Inventory [13]. This measure was then adapted into a carer version (SB-PNI-CV) in the current research, consistent with previous methodology [33] by changing all self-referential terms (i.e. ‘I’) to refer to the relative (i.e. ‘my relative’). The scale operates on a Likert scale from 0 (‘not at all like my relative’) to 5 (‘very much like my relative’). By summing participant responses, a total score of 36 indicates that participants scored on average ‘a little like my relative’ to all questions, indicating the presence of pathologically narcissistic traits. The SB-PNI-CV demonstrated strong internal consistency (α = .80), using all available data (*N* = 1021). Subscales of the measure also demonstrated internal consistency for both grandiose (α = .73) and vulnerable (α = .75) items. Informant-based methods of investigating narcissism and its effects has previously been found to be effective and reliable [30] with consensus demonstrated across multiple observers [38].

### Qualitative analyses

Participants who met inclusion criteria were asked to describe their relative using the Wynne-Gift speech sample procedure as outlined by Gift, Cole [41]. This methodology was developed for interpersonal analysis of the emotional atmosphere between individuals with severe mental illness and their relatives, it has also been used in the context of assessing relational functioning within marital couples [41]. For the purpose of this study, the speech sample prompt was used to elicit descriptive accounts of relational functioning, which included participants responding to the question:

*‘What is your relative like, how do you get on together?’*

Participants were given a textbox to respond to this question in as much detail as they would like. However, participants whose text responses were too brief (< 70 words), were removed from analysis as specified by Gottschalk, Winget [39]. It is important to note however that these participants who were removed (*n* = 399) did not differ from the included participants in any meaningful way regarding demographic information. The mean response length was 233 words (SD = 190) and text responses ranged from 70 to 1279 words.

Analysis of the data occurred in multiple stages. First, a phenomenological approach was adopted which places primacy on understanding the ‘lived experience’ of participant responses [42] whilst ‘bracketing’ researcher preconceptions. This involved reading and re-reading all participant responses in order to be immersed in the participants subjective world, highlighting text passages regarding the phenomenon under examination (i.e. personality features, descriptions of behaviour, etc) and noting comments and personal reactions to the text in the margins. This is done in an attempt to make the researchers preconceptions explicit, in order to attend as close as possible as to the content of what is being said by the participant. Second, codebook thematic analysis was used for data analysis as outlined by Braun, Clarke [43], which combines ‘top down’ and ‘bottom up’ approaches. Using this approach, a theory driven or ‘top down’ perspective was taken [44] in which researchers attempted to understand the reality of participants through their expressed content and within the context of the broader known features informed by the extensive prior work on the topic. In this way, the overarching themes of ‘grandiosity’ and ‘vulnerability’ were influenced by empirically determined features within the research literature (e.g. DSM-5 diagnostic criteria, factors within the PNI), however themes and nodes were free to be ‘split’ or merged organically during the coding process reflecting the ongoing conceptualisation of the data by the researchers. Significant statements were extracted and coded into nodes reflecting their content (e.g. ‘narcissistic rage’, ‘entitlement’) using Nvivo 11. This methodology of data analysis via phenomenologically analysing and grouping themes is a well-documented and regularly utilized qualitative approach (e.g. [45, 46]). Once data analysis had been completed the second author completed coding for inter-rater reliability analysis on 10% of data. The second rater was included early in the coding process and the two reviewers meet on several occasions to discuss the nodes that were included and those that were emerging from the data. 10% of the data was randomly selected by participant ID numbers. At the end of this process, it was then confirmed that the representation of the data also reflected the participant relationships (i.e. marital partner, child etc). Cohen’s Kappa coefficient was used to index inter-rater reliability by calculating the similarity of nodes identified by the two researchers. This method takes into consideration the agreement between the researchers (observed agreement) and compares it to how much agreement would be expected by chance alone (chance agreement). Inter-rater reliability for the whole dataset was calculated as κ = 0.81 which reflects a very high level of agreement between researchers that is not due to chance alone [47].

### Cluster analysis

A cluster analysis dendrogram was generated using Nvivo 11 for purposes of visualisation and to explore the underlying dimensions of the data [48]. This dendrogram displays the measure of similarity between nodes as coded, in which each source (i.e. participant response) is coded by each node. If the source is coded by the node it is listed as ‘1’ and ‘0’ if it is not. Jaccard’s coefficient was used to calculate a similarity index between each pair of items and these items were grouped into clusters using the complete linkage hierarchical clustering algorithm [49].

## Results

Two broad overarching dimensions were identified. The first dimension, titled ‘grandiosity’, included descriptions that were related to an actual or desired view of the self that was unrealistically affirmative, strong or superior. The second dimensions, titled ‘vulnerability’, included an actual or feared view of the self that was weak, empty or insecure. Beyond these two overarching dimensions, salient personality features not accounted for by the ‘grandiose’ or ‘vulnerable’ dimensions were included within a category reflecting ‘other personality features’. Themes not relating specifically to personality style, but that may provide insights regarding character formation or expression were included within the category of ‘descriptive themes’.

A total of 1098 node expressions were coded from participant responses (*n* = 436), with a total of 2182 references. This means participant responses were coded with an average of two to three individual node expressions (e.g. ‘hiding the self’, ‘entitlement’) and there were on average 5 expressions of each node(s) in the text.

### Overarching dimension #1: grandiosity

Participants described the characterological grandiosity of their relative. This theme was made up of ten nodes: ‘Requiring Admiration’, ‘Arrogance’, ‘Entitlement’, ‘Envy’, ‘Exploitation’, ‘Grandiose Fantasy’, ‘Grandiose Self Importance’, ‘Lack of Empathy’, ‘Belief in own Specialness’ and ‘Charming’.

#### Node #1: requiring admiration or attention seeking

Participants described their relative as requiring excessive admiration. For instance, *“He puts on a show for people who can feed his self-image. Constantly seeking praise and accolades for any good thing he does”* (#1256); *“He needs constant and complete attention and needs to be in charge of everything even though he expects everyone else to do all the work”* (#1303).

#### Node #2: arrogance

Relatives were described as often displaying arrogant or haughty behaviours or attitudes. For instance, “*He appears to not be concerned what other people think, as though he is just ‘right’ and ‘superior’ about everything”* (#1476) and *“My mother is very critical towards everyone around her... family, friends, neighbours, total strangers passing by... everybody is ‘stupid’”* (#2126).

#### Node #3: entitlement

Relatives were also described as having a sense of entitlement. For example, *“I paid all of the bills. He spent his on partying, then tried to tell me what to do with my money. He took my bank card, without permission, constantly. Said he was entitled to it”* (#1787) and *“He won’t pay taxes because he thinks they are a sham and he shouldn’t have to just because other people pay”* (#380).

#### Node #4: envy and jealousy

Participants described instances of their relative being envious or jealous of others. Jealousy, being in relation to the threatened loss of important relationships, was described by participants. For instance, after describing the abusive behaviours of their relative one participant stated *“It got worse after our first son was born, because he was no longer the centre of my attention. I actually think he was jealous of the bond that my son and I had”* (#1419). Other participants, despite using the term ‘jealous’, described more envious feelings in their relative relating to anger in response to recognising desirable qualities or possessions of others. For instance, another participant stated *“[they have] resentment for people who are happy, seeing anyone happy or doing great things with their life makes them jealous and angry”* (#1744). Some participants described their relative believing that others are envious of them, for example *“*[*he] thought everyone was jealous he had money and good looks.”* (#979) and *“[he] tried to convince everyone that people were just jealous of him because he had a nice truck”* (#1149).

#### Node #5 exploitation

Relatives were described as being interpersonally exploitative (i.e. taking advantage of others). For instance, one participant stated *“He brags how much he knows and will take someone else’s knowledge and say he knew that or claim it’s his idea”* (#1293). Another participant stated “*With two other siblings that are disabled, she uses funding for their disabilities to her advantage … I do not think she cares much for their quality of life, or she would use those funds for its intended use.” (#998).*

#### Node #6 grandiose fantasy

Participants also described their relatives as engaging in unrealistic fantasies of success, power and brilliance. For instance, the response *“He believes that he will become a famous film screen writer and producer although he has no education in film”* (#1002); *“He was extremely protective of me, jealous and woefully insecure. [He] went on ‘missions’ where he was sure [world war three] was about to start and he was going to save us, he really believes this”* (#1230).

#### Node #7 grandiose self importance

Relatives were described as having a grandiose sense of self-importance (e.g. exaggerating achievements, expecting to be recognised as superior without commensurate achievements). Examples of this include *“He thinks he knows everything … conversations turn into an opportunity for him to ‘educate’ me”* (#1046); “*He tells endless lies and elaborate stories about his past and the things he has achieved, anyone who points out inconsistencies in his stories is cut out of his lif*e” (#178).

#### Node #8 compromised empathic ability

Participants described their relatives as being unwilling to empathise with the feelings or perspectives of others. Some examples include *“she has never once apologized for her abuse, and she acts as if it never happened. I have no idea how she can compartmentalize like that. There is no remorse”* (#1099) and *“[he] is incapable of caring for all the needs of his children because he cannot think beyond his own needs and wants, to the point of his neglect [resulting in] harm to the children”* (#1488).

#### Node #9 belief in own specialness

Relatives were described as believing they were somehow ‘special’ and unique. For example, one participant described their relative as fixated with their status as an *“important [member] of the community”* (#860), another participant stated *“he considers himself a cut above everyone and everything... Anyone who doesn’t see him as exceptional will suffer”* (#449). Other responses indicated their relatives were preoccupied with being associated with other high status or ‘special’ people. For instance, one participant stated that their relative *“likes to brag about how she knows wealthy people as if that makes her a better person”* (#318) and another stating that their relative *“loves to name drop”* (#49).

#### Node #10 charming

Participants also described their relative in various positive ways which reflected their relatives’ likeability or charm. For instance, *“He is fun-loving and generous in public. He is charming and highly intelligent”* (#1401); *“His public persona, and even with extended family, is very outgoing, funny and helpful. Was beloved by [others]”* (#1046) and *“He is very intelligent and driven, a highly successful individual. Very social and personable and charming in public, funny, the life of the party”* (#1800).

### Overarching dimension #2: vulnerability

Participants described the characterological vulnerability of their relative. This theme was made up of nine nodes: ‘Contingent Self Esteem’, ‘Devaluing’, ‘Emotionally Empty or Cold’, ‘Hiding the Self’, ‘Hypersensitive’, ‘Insecurity’, ‘Rage’, ‘Affective Instability’ and ‘Victim Mentality’.

#### Node #1 contingent self esteem

Participants described their relatives as being reliant on others approval in order to determine their self-worth. For instance, *“She only ever seems to be ‘up’ when things are going well or if the attention is on her”* (#1196) and *“He appears to be very confident, but must have compliments and reassuring statements and what not, several times a day”* (#1910).

#### Node #2 devaluing

Relatives were described as ‘putting down’ or devaluing others in various ways and generally displaying dismissive or aggressive behaviours. For instance, *“On more than one occasion, he’s told me that I’m a worthless person and I should kill myself because nobody would care”* (#1078) and *“He feels intellectually superior to everyone and is constantly calling people idiotic, moron, whatever the insult of the day is”* (#1681).

Relatives were also described as reacting to interpersonal disappointment with shame and self-recrimination, devaluing the self. For instance, *“They are extremely [grandiose] … [but] when someone has the confidence to stand up against them they crumble into a sobbing mess wondering why it’s always their fault”* (#1744) and *“I have recently started to stand up for myself a little more at which point he will then start saying all the bad things are his fault and begging forgiveness”* (#274).

#### Node #3 emotionally empty or cold

Participants described regularly having difficulty ‘connecting’ emotionally with their relative. For instance, one participant described that their relative was *“largely sexually disengaged, unable to connect, difficulty with eye contact … he used to speak of feeling dead”* (#1365); another stated *“he was void of just any emotion. There was nothing. In a situation of distress he just never had any feeling. He was totally void of any warmth or feeling”* (#323), another stated *“I gave him everything. It was like pouring myself into an emotional black hole”* (#627).

#### Node #4 hiding the self

Participants reported instances in which their relative would not allow themselves to be ‘seen’, either psychologically or physically. One way in which they described this was through the construction of a ‘false self’. For instance *“He comes across very confident yet is very childish and insecure but covers his insecurities with bullish and intimidating behaviour”* (#2109). Another way participants described this hiding of self was through a literal physical withdrawal and isolation. For example, *“He will also have episodes of deep depression where he shuts himself off from human contact. He will hide in his room or disappear in his sleeper semi-truck for days with no regard for his family or employer”* (#1458).

#### Node #5 hypersensitive

Participants reported feeling as though they were ‘walking on eggshells’ as their relative would respond volatilely to perceived attacks. For instance, *“She cannot take advice or criticism from others and becomes very defensive and abusive if challenged”* (#1485); *“It was an endless mine field of eggshells. A word, an expression would be taken against me”* (#532) and *“Very irrational and volatile. Anything can set her off on a rage especially if she doesn’t get her way”* (#822).

#### Node #6 insecurity

Relatives were described as having an underlying sense of insecurity or vulnerability. For instance *“He really is just a scared little kid inside of a big strong man’s body. He got stuck when he was a child”* (#1481); *“At the core he feels unworthy, like a fake and so pretty much all introspection and self-growth is avoided at all costs”* (#532) and *“At night when the business clothes come off his fears eat him up and he would feel highly vulnerable and needs lots of reassurance”* (#699).

#### Node #7 rage

Participants reported that their relatives were particularly prone to displaying explosive bouts of uncontrolled rage. For example, *“He has a very fragile ego … he will fly off the handle and subject his target to hours of screaming, insults and tantrum-throwing”* (#1078); *“he has a temper tantrum-like rage that is frightening and dangerous”* (#1476); *“He has hit me once. Left bruises on upper arms and back. He goes into rage and has hit walls, hits himself”* (#1637).

#### Node #8 affective instability (symptom patterns)

Relatives were also described as displaying affective instability which may be related to anxiety and depressive disorders. Relatives were commonly described as being ‘anxious’ (#1091) including instances of hypochondria (#1525), agoraphobia (#756), panic (#699) and obsessive compulsive disorder (#2125). Relatives were also commonly described as having episodes of ‘depression’ (#1106) and depressive symptoms such as low mood (#1931), problems sleeping (#1372). Some participants also described their relative as highly suicidal, with suicidality being linked to relationship breakdowns or threats to self-image. For example, *“When I state I can’t take any more or say we can’t be together … he threatens to kill himself”* (#1798); *“If he feels he is being criticised or blamed for something (real or imagined) … his attacks become self-destructive”* (#1800).

#### Node #9 victim mentality

Participants reported that their relatives often described feeling as though they were the victim of attacks from others or taken advantage of in some way. For instance, *“He seems to think that he has been ‘hard done by’ because after all he does for everyone, they don’t appreciate him as much as they should”* (#1476); *“He will fabricate or twist things that are said so that he is either the hero or the victim in a situation”* (#447).

### Other personality features

Participants also reported some descriptions of their relative that were not described within prior conceptualisations of narcissism. This theme was made up of 3 nodes: ‘Perfectionism’, ‘Vengeful’ and ‘Suspicious’.

#### Node #1 perfectionism

Participants repeatedly described their relative displaying perfectionistic or unrelenting high standards for others. For instance, *“I cannot just do anything at home everything I do is not to her standard and perfection*” (#1586) and *“Everything has to be done her way or it’s wrong and she will put you down. She has complete control over everything”* (#1101).

#### Node #2 vengeful

Participants described their relative as being highly motivated by revenge and displaying vindictive punishing behaviours against others. Examples include, *“[He] has expressed thoughts of wanting to hurt those who cause him problems”* (#230); *“He is degrading to and about anyone who doesn’t agree with him and he is very vengeful to those who refuse to conform to his desires”* (#600) and *“Once someone crosses him or he doesn’t get his way, he becomes vindictive and will destroy their life and property and may become physically abusive”* (#707).

#### Node #3 suspicious

Participants described their relative as holding paranoid or suspicious beliefs about others intentions or behaviours. For instance, *“He would start fights in public places with people because he would claim they were ‘looking at him and mimicking him’”* (#1149) and *“She is angry most days, obsessively talking about who wronged her in the past, currently or who probably will in the future”* (#2116).

### Descriptive themes

Several salient descriptive themes were also coded from the data that, while not relating directly to the relatives character, may provide peripheral or contextual information.

#### Descriptive theme #1: trauma

A number of participants described their relative as having experienced a traumatic or troubled childhood. One participant stated that their relatives’ father *“was extraordinarily abusive both emotionally and physically to both him and the mother … [the father] pushed [the relative] as a young boy on prostitutes as a 12th birthday gift … He was beaten on and off from age 6 to 15 when he got tall enough to threaten back”* (#1249). Another participant described the emotional upbringing of their relative *“[his mother was] prone to being easily offended, fighting with him and cutting off all contact except to tell him what a rotten son he was, for months, then suddenly talking again to him as if nothing had ever happened. His father, he said, was strict and expected a lot of him. Both rarely praised him; whenever he accomplished something they would just demand better instead of congratulating him on his accomplishment”* (#1909). Another participant reflected on how their relative’s upbringing may be related to their current emotional functioning, *“personally I think he is so wounded (emotional, physical abuse and neglect) that he had to detach from himself and others so much just to survive”* (#1640).

#### Descriptive theme #2: excessive religiosity

While participant’s comments on their relative’s religiosity were common, the content was varied. Some participants described their relative using religion as a mechanism to control, for instance *“he uses religion in an extremely malignant way. Manipulating verses and religious sayings and interpret them according to his own will”* (#132) and *“very religious. She uses scripture to manipulate people into doing what she wants on a regular basis”* (#1700). One participant described how their relative’s religiosity became infused with their grandiose fantasy *“He has also gone completely sideways into fundamental religious doctrine, as if he knows more than the average ‘Christian’ about End Times, and all kinds of illuminati type conspiracy around that topic. He says God talks to him directly and tells him things and that he has had dead people talk to him”* (#1476). Other participants described how their relative’s religiosity was merely an aspect of their ‘false self’, for example *“she has a wonderful, loving, spiritual facade that she shows to the world”* (#1073).

#### Descriptive theme #3: substance use

Participants regularly described their relative as engaging in substance use. Substances most frequently named were alcohol, marijuana, cocaine and ‘pills’. Participants reported that when their relative was using substances their behaviour often became dangerous, usually through drink driving, one participant stated *“too much alcohol … he would drive back to [his work] … I was always afraid of [a driving accident]”* (#76).

### Subtype expression

Of 436 participants, a total of 348 unique grandiose node expressions were present and a total of 374 unique vulnerable node expressions were present. Of these, 301 participants included both grandiose and vulnerable descriptions of their relative (69% of sample). Only 47 (11% of sample) focused on grandiose features in their description of their relative, and only 88 participants (20% of sample) focused on vulnerable features.

### Cluster analysis

**Fig. 1:**
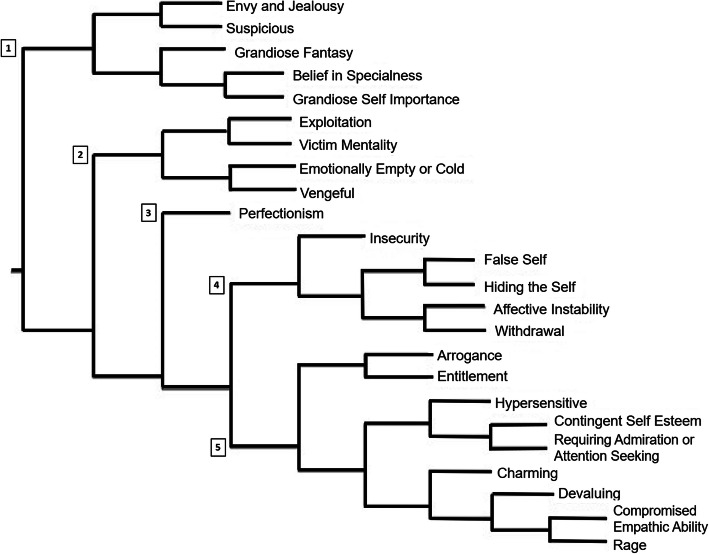
Cluster analysis of nodes based on coding similarity. Note. Clusters are labelled as follows: 1. Fantasy Proneness, 2. Negative Other, 3. Controlling, 4. Fragile Self, 5. Grandiose

A cluster analysis dendrogram was generated using Nvivo 11 for purposes of visualising and exploring the underlying dimensions of the data [48] and is displayed in Fig. 1. Four clusters of nodes and one standalone node can be distinguished. The first cluster, labelled ‘Fantasy Proneness’, includes nodes reflecting the predominance of ‘fantasy’ colouring an individuals interactions, either intrapersonally (‘grandiose self-importance, belief in specialness’) or interpersonally (‘suspicious, envy’). The second cluster, labelled ‘Negative Other’, reflects nodes concerned with a detached connection with others (‘emotionally empty’) and fostering ‘vengeful’ and ‘exploitative’ drives towards others, as well as feelings of victimhood. Interestingly, despite being related to these other aspects of narcissism, ‘perfectionism’ was factored as reflecting its own cluster, labelled ‘Controlling’. The fourth cluster, labelled ‘Fragile Self’, includes nodes indicating feelings of vulnerability (‘affective instability’, ‘insecurity’) and shameful avoidance (‘hiding the self’, ‘false self’, ‘withdrawal’) due to these painful states. The fifth cluster, labelled ‘Grandiose’ reflects a need (‘contingent self-esteem’, ‘requiring admiration’) or expectation (‘entitlement’, ‘arrogance’) of receiving a certain level of treatment from others. It also includes nodes regarding how individuals foster this treatment (‘charming’, ‘rage’, and ‘devaluing’) and a hypervigilance for if their expectations are being met (‘hypersensitive’).

## Discussion

This study aimed to qualitatively describe the interpersonal features of individuals with traits of pathological narcissism from the perspective of those in a close relationship with them.

### Grandiose narcissism

We found many grandiose features that have been validated through empirical research [2, 3, 19]. Grandiosity, as reflected in the DSM-5, has been argued to be a key feature of pathological narcissism that distinguishes it from other disorders [26, 28]. One feature regularly endorsed by participants that is not encompassed in DSM-5 criteria is relatives’ level of interpersonal charm and likability. This charm as described by participants appears more adaptive than a ‘superficial charm’ that might be more exclusively ‘interpersonally exploitative’ in nature. However, it should be noted that this charm did not appear to persist, and was most often described as occurring mainly in the initial stages of a relationship or under specific circumstances (e.g. in public with an audience).

### Vulnerable narcissism

We also found participants described their relative in ways consistent with the vulnerable dimensions of the pathological narcissism inventory (i.e. hiding the self, contingent self esteem and devaluing [50];). Dimensions that are also included in other popular measures for vulnerable narcissism were also endorsed by participants in our sample. For instance, the nodes of ‘hypersensitivity’, ‘insecurity’ and ‘affective instability’ reflect dimensions covered in the Hypersensitive Narcissism Scale [51] and neuroticism within the Five Factor Narcissism Inventory [52]. These aspects of narcissism have also been documented within published literature [12, 27, 53, 54].

### Subtype expression: cluster analysis

Most participants (69% of sample) described both grandiose and vulnerable characteristics in their relative, which given the relatively small amount of text and node expressions provided per participant is particularly salient. Given the nature of the relationship types typically endorsed by participants (i.e. romantic partner, family member), it suggests that the degree of observational data on their relative is quite high. As such, these results support the notion that an individual’s narcissism presentation may fluctuate over time [20, 21] and that vulnerable and grandiose presentations are inter-related and oscillating [9, 19].

The cluster analysis indicates the degree to which salient co-occurring features were coded. These features can be grouped to resemble narcissistic subtypes as described in research literature, such as the subtypes outlined by Russ, Shedler [55] in their Q-Factor Analysis of SWAP-II Descriptions of Patients with Narcissistic Personality Disorder. Our clusters #1–3 (‘Fantasy Proneness’, ‘Negative Other’ and ‘Controlling’) appear to resemble the ‘Grandiose/malignant narcissist’ subtype as described by the authors. This subtype includes instances of self-importance, entitlement, lack of empathy, feelings of victimisation, exploitativeness, a tendency to be controlling and grudge holding. Our cluster #4–5 (‘Fragile Self’ and ‘Grandiose’) appear to resemble the ‘Fragile narcissist’ subtype described including instances of depressed mood, internal emptiness, lack of relationships, entitlement, anger or hostility towards others and hypersensitivity towards criticism. Finally, our ‘Grandiose’ cluster (#5) showed overlap with the ‘high functioning/exhibitionistic narcissist’ subtype, which displays entitled self-importance but also a significant degree of interpersonal effectiveness. We found descriptions of the relative showing ‘entitlement’, being ‘charming’ and ‘requiring admiration’.

While co-occurring grandiose and vulnerable features are described at all levels of clusters in our sample, distinctions between the observed clusters may be best understood as variations in level of functioning, insight and adaptiveness of defences. As such, pathological narcissism has been understood as a characterological way of understanding the self and others in which feelings of vulnerability are defended against through grandiosity [56], and threats to grandiosity trigger dysregulating and disintegrating feelings of vulnerability [53]. Recent research supports this defensive function of grandiosity, with Kaufman, Weiss [11] stating *“*grandiose narcissism was less consistently and strongly related to psychopathology … and even showed positive correlations with adaptive coping, life satisfaction and image-distorting defense mechanisms*”* (p. 18). Similarly, Hörz-Sagstetter, Diamond [28] state ‘high levels of grandiosity may have a stabilizing function’ on psychopathology (p. 569). This defence, however, comes at a high cost, whether it be to the self when the defensive grandiosity fails (triggering disintegrating bouts of vulnerability) or to others, as this style of relating exacts a high toll on those in interpersonal relationships [33].

### Other personality features

Participants described their relative as highly perfectionistic, however the perfectionism described was less anxiously self-critical and more ‘other oriented’. This style of other oriented ‘narcissistic perfectionism’ has been documented by others [57] and appears not to have the hallmarks of overt shameful self-criticism at a surface level, however may still exist in covert form [58]. Regarding the ‘vengeful’ node, Kernberg [16], Kernberg [59] describes that as a result of a pain-rage-hatred cycle, justification of revenge against the frustrating object is an almost unavoidable consequence. Extreme expressions of acting out these “ego-syntonic” revenge fantasies may also highlight the presence of an extreme form of pathological narcissism in this sample – malignant narcissism, which involves the presence of a narcissistic personality with prominent paranoia and antisocial features [60]. Lastly, Joiner, Petty [61] report that depressive symptoms in narcissistic personalities may evoke paranoid attitudes, which may in turn be demonstrated in the behaviours and attitudes expressed in the ‘suspicious’ node we found.

While this study focused on a narcissistic presentation, the presence in this sample of these other personality features (which could alternatively be described as ‘anankastic’, ‘antisocial’ and ‘paranoid’) is informed by the current conversation regarding dimensional versus categorical approaches [62, 63]. Personality dysfunction from a dimensional perspective, such as in the ‘borderline personality organisation’ [23] or borderline ‘pattern’ [64] could understand these co-occurring personality features as not necessarily aspects of narcissism or ‘co-morbidities’, but as an individual’s varied pattern of responding that exists alongside their more narcissistic functioning, reflecting a more general level of disorganisation that resists categorisation. This is particularly reflected in Table 2 as participants reported a wide variety of diagnosed conditions, as well as the ‘Affective Instability’ node which may reflect various diagnostic symptom patterns.

### Descriptive features

The relationship between trauma and narcissism has been documented [58, 65–67] and the term ‘trauma-associated narcissistic symptoms’ has been proposed to identify such features [68]. Interestingly, while participants in our sample did describe instances of overt abuse which were traumatic to their relative (e.g. physical, verbal, sexual), participants also described hostile environments in which maltreatment was emotionally abusive or manipulative in nature, as well as situations where there was no overt traumatic abuse present but which most closely resemble ‘traumatic empathic failures’. This type of attachment trauma, stemming from emotionally invalidating environments, is central to Kohut’s theory of narcissistic development [69, 70], and has found support in recent research [71]. Relatives religiosity was noteworthy, not necessarily due to its presence, but due to the narcissistic function that the religiosity served. Research on narcissism and religious spirituality has steadily accumulated over the years (for a review see: [72]) and the term ‘spiritual bypassing’ [73] is used for individuals who use religion in the service of a narcissistic defence. In our sample this occurred via alignment with an ‘ultimate authority’ in order to bolter esteem and control needs. It may be that the construction of a ‘false self’ rooted in spirituality is conferred by the praise and audience of a community of believers. Finally, participants reported their relative as engaging in various forms of substance use, consistent with prevalence data indicating high co-occurrence of narcissism and substance use [65]. While the motivation behind relatives substance use was not mentioned by participants, it is consistent with relatives more general use of reality distorting defences, albeit a more physicalised as opposed to an intrapsychic method.

### Implications of findings

First, this study extends and supports the widespread acknowledged limitation of DSM-5 criteria for narcissistic personality disorder regarding the exclusion of vulnerable features (for a review of changes to dignostic criteria over time, see [74, 75]) and we acknowledge the current discussion regarding therapist decision to provide a diagnosis of NPD [76]. However, the proliferation of alternate diagnostic labels may inform conceptualisations which do not account for the full panorama of an individual’s identity [7], adding to the already contradictory and unintegrated self-experience for individuals with a narcissistic personality. This may also impede the treatment process by informing technical interventions which may be contra-indicated. For instance, treatment of individuals with depressive disorders require different approaches than individuals with a vulnerably narcissistic presentation [24, 77]. As such, a focus of treatment would include the integration of these disparate self-experiences, through the exploration of an individual’s affect, identity and relationships, consistent with the treatment of personality disorders more generally. Specifically, when working with an individual with a narcissistic personality, this may involve identifying and clarifying instances of intense affect, such as aggression and envy, themes of grandiosity and vulnerability in the self-concept, and patterns of idealization and devaluation in the wider relationships. The clinician will need to clarify, confront or interpret to these themes and patterns, their contradictory nature as extreme polarities, and attend to the oscillation or role reversals as they appear [78]. Second, as the characterological themes identified in this paper emerged within the context of interpersonal relationships, this highlights the interconnection between impaired self and other functioning. As such, in the context of treating an individual with pathological narcissism, discussing their interpersonal relationships may be a meaningful avenue for exploring their related difficulties with identity and emotion regulation that may otherwise be difficult to access. This is particularly salient as treatment dropout is particularly high for individuals with pathological narcissism [4], and as typical reason for attending treatment is for interpersonal difficulties [79]. Third, treatment for individuals with narcissistic personalities can inspire intense countertransference responses in clinicians [80] and often result in stigmatisation [81]. As such, these findings also provide a meaningful way for the clinician to extend empathy to these clients as they reflect on the defensive nature of the grandiose presentation, the distressing internal emptiness and insecurity for these individuals, and the potential childhood environment of emotional, sexual or physical trauma and neglect which may have informed this defensive self-organisation. Finally, these findings would also directly apply to clinicians and couples counsellors working with individuals who identify their relative as having significant narcissistic traits, providing them with a way to understand the common ways these difficulties express themselves in their relationships and the impact they may have on the individuals in the relationship. Practically, these findings may inform a heightened need for treating clinicians to assess for interpersonal violence and the safety of clients in a context of potential affective dysregulation and intense aggression. Regarding technical interventions, if working with only one of the individuals in the relationship, these findings may provide avenues for psychoeducation regarding their relatives difficulties with identity and affect regulation, helping them understand the observed oscillating and contradictory self-states of their relative. If working with both individuals or the couple, the treating clinician will need to be able to identify and interpret changes in affect and identity, and the way this manifest in the relationship functioning of the couple and their characteristic ways of responding to each other (e.g. patterns of idealization and devaluation). This may also involve attending to the ways in which the therapist may be drawn into the relationship with the couple, noticing and interpreting efforts at triangulation or any pressure to ‘pick sides’ from either individual.

### Limitations

The sample selection procedure may have led to results only being true for some, but not all people living with a relative with narcissistic features. Participants were recruited online limiting the opportunity to understand participant motivation. Second, relying on informant ratings of narcissism for both screening and qualitative analysis is a limitation as we are less unable to control for severity, specificity or accuracy of participant reporting. Further, it is possible that the use of a narcissism screening tool primed participants to artificially report on particular aspects of their relative. However, the risk of biasing or priming participants is a limitation of all studies of this kind, as studies implementing informant methodology for assessing narcissism typically rely on providing participants with a set of diagnostic criteria or narcissism specific measures as their sole indicator of narcissism (e.g. [30, 38]). As such, notwithstanding the limitations outlined, this informs the novelty and potential utility of the present approach which relies on identifying narcissism specific features amongst a backdrop of descriptions of more general functioning within intimate relationships. Third, gender disparity in participants and relatives was substantial. However, as NPD is diagnosed more commonly in males (50–75%, American Psychiatric Association, 2013) and as most participants in our sample were in a romantic, heterosexual relationship, this disparity may reflect a representative NPD sample and should not significantly affect the validity of results. Rather, this disparity may strengthen the argument that individuals with a diagnosis of NPD (as specified by DSM-5 criteria) may have co-occurring vulnerable features, which may not be currently reflected in diagnostic categories. Finally, as a result of relying on informant ratings and not assessing narcissistic individuals via structured clinical interview, questions regarding the specificity and severity of the narcissistic sample are unable to be separated in the analysis. We thus probably studied those ranging from ‘adaptive’ or high functioning narcissism [82] to more severe and disabling character disorders. Whilst we screened for narcissistic features, it was clear the sample studied also reported a broad range of other co-occurring problems.

## Conclusions

We investigated the characteristics of individuals with pathologically narcissistic traits from the perspective of those in a significant personal relationship with them. The overarching theme of ‘Grandiosity’ involved participants describing their relative as requiring admiration, displaying arrogant, entitled, envious and exploitative behaviours, engaging in grandiose fantasy, lacking in empathy, having a grandiose sense of self-importance, believing in own sense of ‘specialness’ and being interpersonally charming. The overarching theme of ‘Vulnerability’ involved participants describing their relative’s self-esteem being contingent on others, as being hypersensitive, insecure, displaying affective instability, feelings of emptiness and rage, devaluing self and others, hiding the self through various means and viewing the self as a victim. Relatives were also described as displaying perfectionistic, vengeful and suspicious personality features. Finally, participants also described several descriptive themes, these included the relative having a trauma history, religiosity in the relative and the relative engaging in substance use. The vulnerability themes point to the problems in the relatives sense of self, whilst the grandiose themes show how these express themselves interpersonally. The complexity of interpersonal dysfunction displayed here also points to the importance of assessing all personality traits more broadly.

## Data Availability

The datasets generated during and/or analysed during the current study are not publicly available due to the sensitive and personal nature of participant responses but are available from the corresponding author on reasonable request.
