# Correction to: Living with pathological narcissism: a qualitative study

**DOI:** 10.1186/s40479-022-00177-x

**Published:** 2022-01-22

**Authors:** Nicholas J. S. Day, Michelle L. Townsend, Brin F. S. Grenyer

**Affiliations:** Illawarra Health and Medical Research Institute and School of Psychology, University of Wollongong Australia, Wollongong, NSW Australia

**Correction to: Borderline Personal Disord Emot Dysregul 7, 19 (2020).**

**https://doi.org/10.1186/s40479-020-00132-8**

Following publication of this article [1], it is reported that the reference 35 contained an error.

The correct reference 35 should be:

35. Green A, Charles K. Voicing the Victims of Narcissistic Partners: A Qualitative Analysis of Responses to Narcissistic Injury and Self-Esteem Regulation. SAGE Open. 2019;9(2). 10.1177/2158244019846693.

The original article has been updated.
